# Bilateral Giant Juvenile Fibroadenomas of Breasts:A Case Report

**DOI:** 10.4061/2011/482046

**Published:** 2011-05-30

**Authors:** D. B. Nikumbh, S. R. Desai, P. S. Madan, N. J. Patil, J. V. Wader

**Affiliations:** Department of Pathology, Krishna Institute of Medical Sciences, Karad, District Satara, Maharashtra 415110, India

## Abstract

Juvenile fibroadenoma constitutes only 4% of the total fibroadenomas. The incidence of giant juvenile fibroadenomas is found to be only 0.5% of all the fibroadenomas. Bilateral giant juvenile fibroadenomas are extremely rare, and only four cases have been reported in the literature. To the best of our knowledge, we are presenting the fifth case of bilateral giant juvenile fibroadenomas in a 12-year-old prepubertal girl. The diagnosis was made on fine-needle aspiration cytology which was confirmed on histopathology. In this paper, we present this rare case to illustrate the diagnosis and management of this tumour and to emphasize that these tumours are almost always benign and should be treated with breast-conserving surgery to provide a healthy physical and social life to the patient.

## 1. Introduction

Breast masses are uncommon in childhood. The majority of them are related to inflammation (infection or abscess) [1] or benign tumors as fibroadenomas [2]. Juvenile fibroadenoma is a rare clinical entity and forms 4% of thetotal fibroadenomas, and giant juvenile fibroadenoma constitutes only 0.5% of all fibroadenomas [3, 4]. Bilateral giant juvenile fibroadenomas are extremely rare in prepubertal girls. To the best of our knowledge, only four case reports are available in the English literature [3, 5–7]. Stanford School of Medicine clearly defined both the entities of juvenile and giant fibroadenoma [8]. Our case satisfied both these definitions; therefore, it is a rare case of bilateral giant juvenile fibroadenomas in a twelve-year-old prepubertal female child. The provisional diagnosis of the patient was made on fine-needle aspiration cytology which was subsequently confirmed on histopathology.

## 2. Case Report

**Figure 1:**
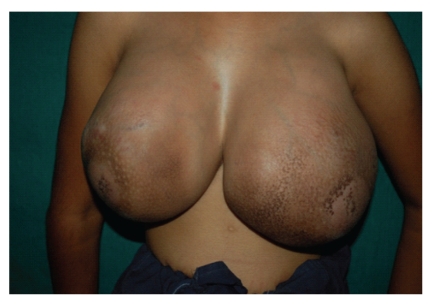
Gross appearance of bilateral breasts tumors.

**Figure 2:**
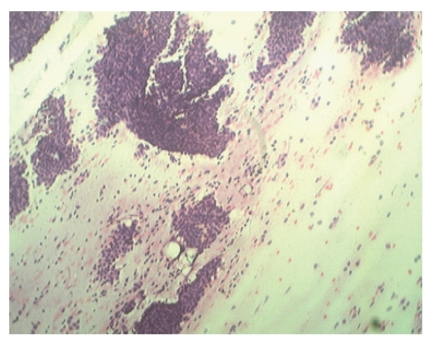
FNAC from breast lumps showed many branching sheets of epithelial cells with many bare nuclei and myxoid stromal fragments in background. (H &E stain X100).

A 12-year-old premenarche girl presented with bilateral, rapidly enlarging breast lumps for three months (Figure 1). There was history of dull ache in the breasts. There was no family history. History of trauma, nipple discharge, fever, anorexia, or weight loss was absent. On local examination bilateral slightly tender, huge, well-circumscribed masses in both the breasts were seen, which were firm in consistency. The overlying skin was tense and shiny with prominent superficial veins. The right breast lump measured 15 × 12 cms and left breast lump measured 17 × 15 cms. On both the sides, the lumps were not fixed to underlying structures. There was no discharge from the nipple, and axillary lymphadenopathy was absent. Routine hematological and biochemical examinations were within normal limits. Chest-X ray was normal. Ultrasonography was carried out which showed heterogenous parenchymal pattern suggestive of bilateral fibroadenoma. The patient was subjected to fine-needle aspiration cytology (FNAC) of bilateral breast lumps which revealed cellular smears comprising of many branching monolayer sheets along with myoepithelial cells (Figure 2).

**Figure 3:**
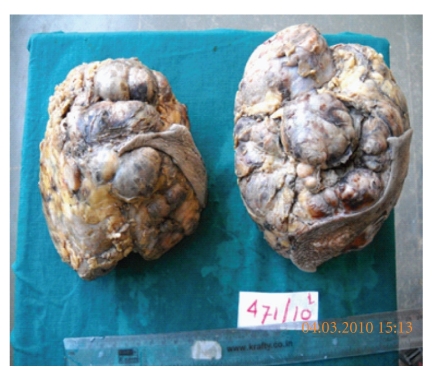
Gross appearance of bilateral lumpectomy specimens.

**Figure 4:**
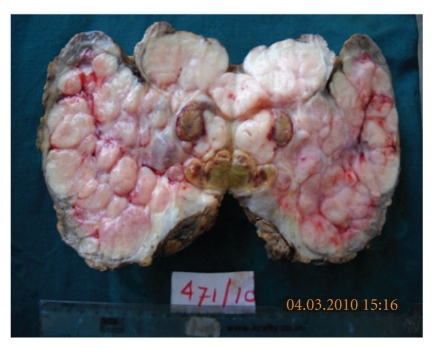
Cut surface of both the specimens showed multiple gray white nodules with myxoid appearance and occasional slit-like spaces.

Background showed bare nuclei and myxoid stromal fragments. Based on the cytological findings, diagnosis of benign proliferative lesion without atypia suggestive of bilateral fibroadenomas was given. The patient underwent total excision of bilateral breast lumps conserving the normal breast tissue, nipple, and areola by the plastic surgeon. The two resected specimens were sent for histopathological study in the department of pathology. The right-sided excised lump measured 15 × 12 × 6.5 cms and weighed 800 gms. The left-sided excised lump measured 17 × 15 × 5 cms and weighed 950 gms. Overlying skin was unremarkable. External surface was bosselated, and both the lumps were well circumscribed and encapsulated (Figure 3). Cut sections from both the lumps showed multiple gray white nodules showing myxoid, gray white appearance along with occasional slit-like spaces and tiny cysts (Figure 4).

## 3. Light Microscopic Findings

**Figure 5:**
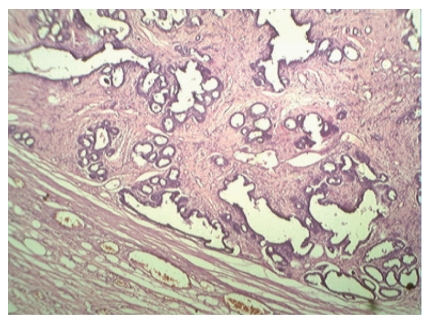
Light microscopy showed well-encapsulated tumor with hyperplasia of both epithelial and stromal components. (H&E stain X100).

**Figure 6:**
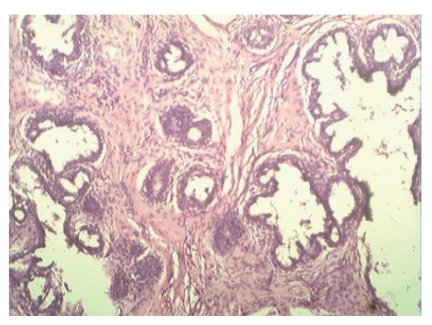
Light microscopy revealed many glands lined by bilayered epithelium at places showed stratification and cellular stoma with myxoid change (H&E Stain X400).

Hematoxylin- and eosin-stained sections revealed well-encapsulated tumors with hyperplasia of both epithelial and stromal components. Epithelial component showed tubular or tufted pattern of epithelial hyperplasia with characteristic tufts of cells protruding into the lumina (Figure 5). The glands were lined by bilayered epithelium at places showing stratification. Most of the areas in both the tumors showed a pericanalicular pattern of fibroadenoma. Stroma was cellular and showed myxoid change (Figure 6). Though stromal cellularity was increased, there was no leaf-like growth pattern and focal periductal concentrate of cells which was the characteristic of phyllodes tumor. There was occasional mitosis (0-1/hpf) and absence of cytological atypia. The final histopathological diagnosis of Bilateral Giant Juvenile Fibroadenomas of breasts was given. The patient is on regular followup and doing well at six-month followup.

## 4. Discussion

 According to Stanford School of Medicine, juvenile fibroadenoma of the breast is defined as circumscribed, often large, breast mass usually occurring in adolescent females with stromal and epithelial hypercellularity but lacking the leaf-like growth pattern of phyllodes tumors [8]. Diagnostic criteria for juvenile fibroadenoma are (1) circumscribed and rarely multiple; (2) biphasic stromal and epithelial process in which pericanalicular pattern is most common and lacks leaf-like growth pattern in uniformly hypercellular stroma. Fibrotic areas may be present; (3) lack of atypical features in stroma-like periductal increase in cellularity, stromal overgrowth, cytologic atypia, and mitotic rate >3/hpf; (4) frequent epithelial and myoepithelial hyperplasia; (5) most patients' age is 10–20 years with a mean age of 15 years. Juvenile fibroadenomas may be multiple [8]. 

Giant fibroadenoma is defined as a tumor >500 gms or disproportionally large compared to the rest of the breast. It is more frequently seen in young and black patients. Giant fibroadenoma may be either adult type or juvenile fibroadenoma [8, 9]. 

Giant juvenile fibroadenoma simultaneously occurring in both the breasts is very rare. Four case reports are available in the English literature [3, 5–7]. Giant juvenile fibroadenoma is an uncommon tumor presenting in adolescent females and the exact etiology is not known. Hormonal influences are thought to be contributing factors [9]. Excessive estrogen stimulation and/or receptor sensitivity or reducedlevels of estrogen antagonist during puberty have been implicated in pathogenesis [9, 10].

It is necessary to exclude the close differentials of juvenile fibroadenoma which are benign low-grade phyllodes tumor, virginal hypertrophy, and other rare differentials such as lipoma, hamartoma, breast abscess, macrocyst, adenocarcinoma, and pseudoangiomatous stomal hyperplasia, as the treatment modalities and the prognosis differ quite significantly in these various conditions. Some of the lesions were treated by mastectomy, but some lesions may require only local excision, aspiration, or conservative management [10–12].

Giant juvenile fibroadenoma is a benign tumor, and total excision of the lump with conservation of nipple and areola is the optimal treatment [10–12]. 

Benign low-grade phyllodes tumor occurs in the older (>40 years) age group and has no racial predisposition. Generally, they present as a solitary mass confined to unilateral breast, and bilateral involvement is rarely seen [10]. In low-grade phyllodes tumor, prominent leaf-like architecture is seen due to stromal cellularity and, there is a characteristic stromal condensation around the ducts, and it infiltrates the surrounding breast tissue with mitotic figures <4/hpf. In high-grade phyllodes tumor, stromal overgrowth with atypia and atypical mitotic figures (<10/hpf) are seen. It is treated by wide excision with a margin of normal tissue or mastectomy [10, 11, 13, 14].

In juvenile breast hypertrophy, rapid and distressing enlargement of one or both breasts occurs, which is often asymmetrical and occasionally in an adolescent female. Histological examination shows abundant connective tissue and duct proliferation, frequently with epithelial hyperplasia but little or no lobule formation. It is treated by reduction mammoplasty [10, 12, 14].

Giant lipoma can cause unilateral breast hypertrophy. Soft, mobile mass can be felt on palpation. Lipoma consists of encapsulated nodules of mature adipose tissue on histology [10, 12, 14].

Breast abscesses developing during puberty cause sudden and rapid growth in the breast. Pain, fluctuation, and erythema are present. Histological examination reveals focal collection of polymorphs with necrotic material in the lobules. Hamartomas can be easily suspected on imaging with their multilobular structures. Microscopically, these lesions are composed of an admixture of ducts, lobules, fibrous stroma, and adipose tissue in varying proportions [13, 14]. Pseudoangiomatous stromal hyperplasia reveals complex interanastomosing spaces, some of which have spindle-shaped stromal cells at their margins simulating endothelial cells [10, 12, 14]. FNAC and biopsy easily rule out these conditions.

It is essential to know that giant juvenile fibroadenoma may recur after complete excision, and the chance of recurrence becomes less after the third decade [13]. In view of bilateral giant juvenile fibroadenomas, possibility of Carneys complex should be considered, which constitutes multiple myxoid fibroadenomas, endocrine hyperactivity, cardiac myxoma, cutaneous hyperpigmentation, and other abnormalities [14].

In our case, other components of Carneys complex were absent with the lack of family history except for multiple fibroadenomas.

Isolated case reports ofunilateral juvenile fibroadenoma and multiple giant fibroadenoma in single breast were available [9, 12, 15, 16]. In the literature, only four case reports of bilateral giant juvenile fibroadenomas were reported [3, 5–7]. The last case was reported in 2009 [7]. To conclude, we present an extremely rare case of bilateral giant juvenile fibroadenomas of breasts. The case was diagnosed on FNAC and subsequently confirmed on histopathology. The patient is treated by the removal of both the fibroadenomas conserving the breast tissue. The patient is doing well with regular followup.
